# Caffeine reduces hepatic lipid accumulation through regulation of lipogenesis and ER stress in zebrafish larvae

**DOI:** 10.1186/s12929-015-0206-3

**Published:** 2015-11-17

**Authors:** Xinchun Zheng, Wencong Dai, Xiaohui Chen, Kunyuan Wang, Wenqing Zhang, Li Liu, Jinlin Hou

**Affiliations:** State Key Laboratory of Organ Failure Research, Guangdong Provincial Key Laboratory of Viral Hepatitis Research, Department of Infectious Diseases, Nanfang Hospital, Southern Medical University, Guangzhou, 510515 China; Key Laboratory of Zebrafish Modeling and Drug Screening for Human Diseases of Guangdong Higher Education Institutes, Department of Cell Biology, Southern Medical University, Guangzhou, 510515 China

**Keywords:** Caffeine, Zebrafish, Fatty liver, ER stress, Lipogenesis

## Abstract

**Background:**

Caffeine, the main component of coffee, has showed its protective effect on non-alcoholic fatty liver disease (NAFLD) in many studies. However, the hepatoprotection of caffeine and its mechanisms in zebrafish were unexplored. Thus, this study’s intentions are to establish a NAFLD model of zebrafish larvae and to examine the role of caffeine on fatty liver with the model.

**Results:**

Growth and the incidence of fatty liver of zebrafish larvae increased with the increased amount of feeding in a dose-dependent manner. The degree of hepatic steatosis of larvae also gradually aggravated with the increased quantity and duration of feeding. Triglyceride contents of zebrafish fed for 20 days significantly increased in model group (180 mg/d) compared with control group (30 mg/d) (*P* < 0.001). Significant decreases in body weight and hepatic steatosis rate were observed in 2.5, 5, 8 % caffeine treatment group compared with model group (*P* < 0.05). Hepatic lipid accumulation was also significantly reduced in caffeine treatment larvae. Moreover, caffeine treatment was associated with upregulation of lipid β-oxidation gene ACO and downregulation of lipogenesis-associated genes (SREBP1, ACC1, CD36 and UCP2), ER stress-associated genes (PERK, IRE1, ATF6 and BIP), the inflammatory cytokine genes (IL-1beta and TNF-alpha) and autophagy associated genes (ATG12 and Beclin-1). Protein expression of CHOP, BIP and IL-1beta remarkably reduced in caffeine treatment group compared with model group.

**Conclusions:**

We induced hepatoteatosis in zebrafish by overfeeding regimen and demonstrated caffeine have a role in suppression of hepatosteatosis by downregulation of genes associated with lipogenesis, ER stress, inflammatory response and enhancement of lipid oxidation, indicating zebrafish model may be used to identify putative pharmacological targets and to test novel drugs for human NAFLD treatment.

## Background

NAFLD is becoming one of the most common causes of chronic liver diseases. It is estimated that approximately 20–30 % of adults and 3–10 % of children in western countries suffer from excessive fat accumulation in the liver [1]. Following the prevalence of obesity and its associated complications such as diabetes, insulin resistance and hyperlipidemia in the world, the incidence of NAFLD have been increasing in recent years. Indeed, NAFLD is considered to be a manifestation of metabolic syndrome in the liver [2, 3], which range from non-progressive simple steatosis to non-alcoholic steatohepatitis (NASH) with ballooning degeneration, inflammation and fibrosis. NASH can progress from steatohepatitis to liver cirrhosis, and may eventually develop hepatocellular carcinoma after decade years.

There are many studies that have attempted to establish NAFLD models in rodents via the use of dietary [4, 5] and pharmacological [6] induction, as well as genetic manipulation [7, 8]. Recently, zebrafish compare popularly to rodents as new experimental animals for researchers because zebrafish have a high reproductive rate, mature rapidly, and cost little regard to rearing space and daily maintenance due to their small size. More and more researchers use zebrafish as a model to study the pathogenesis and pharmacological therapy of NAFLD. In a review, Yoichi Asaoka et al. [9] summarized the various types of fish models in use for NAFLD, including those generated by mutation [10], transgenesis [11–15], or dietary [16, 17] or chemical [18, 19] treatment, and contrast them with rodent models. And recently Valerie Sapp et al. [20] have established a fructose-induced zebrafish model of NASH in 7 days post-fertilization (dpf) zebrafish larvae. However, these previous study for NAFLD using zebrafish model mostly focused on the period of 5dpf-7dpf zebrafish larvae and adult zebrafish, there is lack of knowledge about studying juvenile zebrafish larvae of NAFLD. Because of the advantage of transparent body in juvenile period suitable for identifying lipid accumulation in liver by whole-mount oil red O staining, in present study we attempted to determine whether juvenile zebrafish can be used as a model for diet-induced NAFLD.

Coffee is probably the most frequently consumed beverage worldwide. Because of its consumption in most countries in the world, it is concerned to investigate its potential benefits or adverse aspects in relationship to human health in terms of a public and a scientific perspective. Caffeine is a main component of coffee, which also includes other ingredients such as diterphenoic alcohols, potassium, niacin, magnesium, and the anti-oxidants chlorogenic acid (CGA) and tocopherols. Although coffee and caffeine can increase the risk of cardiovascular disease, numerous evidences demonstrated that coffee and caffeine have a hepatoprotective effect on chronic liver diseases [21, 22]. Epidemiological and clinical investigations have suggested that its consumption could reduce the risk of alcoholic liver cirrhosis [23], type 2 diabetes [24–26], NAFLD [27–29], and HCC [30–33] and the progression of NASH and the severity of fibrosis [34], as well as the activity of ALT in liver injury patients [35]. There are many studies that show coffee and caffeine exhibit a beneficial role on liver injury in animal and in vitro studies. Sandra kal thoff et al. have demonstrated that coffee mediate the protective and antioxidant properties through upregulation of expression of glucuronosyltransferases in liver and stomach [36]. Experimental study has shown that caffeine can attenuate the development of liver cirrhosis and HCC through inhibition of transforming growth factor-beta (TGF-beta) and its downstream effectors [37]. Recent study has demonstrated that caffeine reduces intrahepatic lipid content and stimulates β-oxidation in hepatic cells and liver by an autophagy-lysosomal pathway via using genetic, pharmacological, and metabolomic approaches [38]. Similarly, researchers have shown that caffeine effectively depletes TG and cholesterol levels by inhibition of lipogenesis and stimulation of lipolysis through modulating AMPK-SREBP signalling pathways in HepG2 cells [39]. While coffee and caffeine show a hepatoprotective characteristic on NAFLD, the precise mechanism is completely unknown.

In this study, we determined the effect of caffeine on lipid accumulation in the liver of zebrafish larvae by establishment of diet-induced model of NAFLD in juvenile zebrafish. We have developed a zebrafish model for NAFLD and confirmed the model by morphological, chemical and histological analyses. Our results shown that caffeine can reduce hepatic lipid accumulation in diet-induced zebrafish, which probably exerts antisteatotic effect through reduced input of fatty acid and inhibition of lipogenesis and improvement of ER function, and attenuation of inflammation response.

## Methods

### Feeding zebrafish

Wild-type zebrafish (Danio rerio) were raised and cared for as per standard procedures in accordance with a protocol approved by the Southern Medical University Animal Care and Use Committee. Embryos and larvae were reared in embryo medium at 28 °C to 5 dpf. Zebrafish larvae with completely development from a single cluster at 5dpf were allocated randomly to a serial of different amount of feeding groups: 20 mg/d, 30 mg/d, 60 mg/d, 80 mg/d, 120 mg/d and 180 mg/d. Each group had 100 larvae and started to feed at 7 dpf for 10, 15 and 20 days in 1.5 L tanks after adaptive of deep water environment for one day. They were fed a larval food (Zeigler AP100) three times daily, which was grinded to powder until food can float on the face of water, and then exchanged by half water after fed for 2 h. For caffeine treatment, larvae were overfed with 1, 2.5, 5 and 8 % caffeine for 20 days, which were mixed well with larval food (Zeigler AP100) and grinded to powder. At each time point, all larvae in a tank were counted and harvested and killed by tricane (Sigma) overdose. The length of zebrafish was measured from tip of the nose to the end of body. Larvae were wiped dry and weighed on a Mettler AE 50 balance to the nearest milligram.

### Biochemical analyses of zebrafish lipids

Zebrafish larvae (*n* = 15–20 each) were homogenized and diluted 10 times with double distilled water, centrifuged and got supernatant liquid. Levels of total cholesterols (TCH) and triglycerides (TGs) in the supernatant were determined by Total Cholesterol Reagent Kit and Triglyceride Reagent Kit (ZheJiang DongGou Diagnostics Co.,LTD, China) according to the manufacturer’s specification, respectively.

### Food intake of zebrafish larvae

20 dpf zebrafish larvae were allocated to normal feeding and caffeine feeding group. Each group which have 15 larvae have a similar body weight and were fed with 20 mg food in 100 ml tanks. After feeding 2 h, the remainder of food in water and on the bottom of tank were collected, dried and weighted. Then the food intake of 15 larvae was calculated by the feeding quantity minus the remainder amount.

### Whole-mount oil red O staining

Zebrafish larvae were fixed by 4 % paraformaldehyde solution in PBS overnight 4 °C for 3 days, washed in PBS and orderly immersed in 40, 60, 80 and 100 % 1,2-propanediol at room temperature for 20 min respectively, and then washed in PBS. Added fresh 0.5 % Oil Red O solution, larvae were dyed at room temperature for 12 h, and washed in PBS, and faded background color by adding 100 % 1,2-propanediol. Stained larvae were stored in 80 % 1, 2-propanediol and then imaged with a bright-field dissecting microscope (Olympus cellSens). Larvae were defined as positive for steatosis if the boundary between liver and surrounding tissue is clear and more than three lipid droplets were observed in the hepatic paranchyma by staining.

### Histologic assessment

Zebrafish were fixed using 4 % paraformaldehyde solution in PBS overnight at 4 °C, washed in PBS and equilibrated in 30 % sucrose/PBS overnight at 4 °C. Then they were embedded in OCT and cut using a cryostat for 8 μm section. Consecutive sections were paired on adjacent slides; one slide in each pair was stained with oil red O and the other in hematoxylin and eosin (HE). For frozen oil red O staining, after drying the sections were immersed sequentially in 100, 85 % 1, 2 –propanediol for 5 min. The sections were then immersed in a 0.5 % Oil Red O (Sigma) solution for 2 h and rinsed with distilled water. Sections were then counterstained using hematoxylin to visualize the nuclei. As for frozen HE staining, the sections were treated sequentially to 100, 95, 90, 85, 80,70 % ethanol, and stained with hematoxylin,washed in running tap water,stained with eosin,washed with running tap water, and mounted using aqueous mounting media, Glycerin Jelly.

### Quantitative real-time reverse transcriptase polymerase chain reaction (qRT-PCR)

Total RNA samples from 20 zebrafish larvae liver were used to generate cDNA using PrimeScript^TM^ RT reagent Kit with gDNA Eraser (Perfect Real Time) (Takara, Japan). QRT-PCR was done using a Roche LightCycler480 System with FastStart Essential DNA Green Master (Roche, USA). The thermal cycling condition comprised an initial step at 95 °C for 1 min followed by 40 cycles of 95 °C for 10 s, 60 °C for 20 s and 72 °C for 30 s. The primers used in this study are shown in Table 1. The mRNA level of liver samples in all groups was normalized using expression of eukaryotic translation elongation factor 1 alpha 1, like 1 (EFL1-alpha) as a housekeeping gene and was relative to control group according to the 2^−ΔΔCT^ method. Each sample was analysed in triplicate.

**Table 1:** Primer sequences used for quantitative real-time PCR (qPCR)

Gene	Accession	Forward primer	Reverse primer
PPAR-γ	DQ839547	5′-GGTTTCATTACGGCGTTCAC-3′	5′-TGGTTCACGTCACTGGAGAA-3′
SREBP1	NM_001105129	5′-CATCCACATGGCTCTGAGTG-3′	5′-CTCATCCACAAAGAAGCGGT-3′
PPAR-α	NM_001102567	5′-CGTCGTCAGGTGTTTACGGT-3′	5′-AGGCACTTCTGGAATCGACA-3′
ACC1	XM_009298360	5-′GCATAGGGCAGGTTTTACCA-3′	5′-GCCATCATACGAGAGCAACA-3′
FASN	XM_009306807	5-′GAGAAAGCTTGCCAAACAGG-3′	5′-GAGGGTCTTGCAGGAGACAG-3′
CD36	NM_001002363	5′-AGGCCACTGTGAACCTGAAG-3′	5′-AAGTTGGGGTTCATTCCGAC-3′
UCP2	NM_131176	5′-GAGCTTTGCTTCTGTACGCA-3′	5′-ACGAAACCCCTCTTCCTTTG-3′
CPT I	NM_001044854	5′-ACTCTCGATGGACCCTGTGA-3′	5′-CTGGATGAAGGCATCTGGAC-3′
ACO	NM_213147	5′-AAGGACATCGAGCGAATGAT-3′	5′-CTATGAAAGAGTGGAGGCCG-3′
ATF6	NM_001110519	5′-CTGTGGTGAAACCTCCACCT-3′	5′-CATGGTGACCACAGGAGATG-3′
IRE1	XM_009306258	5′-TGACGTGGTGGAAGTTGGTA-3′	5′-ACGGATCACATTGGGATGTT-3′
PERK	XM_005156585	5′-TGGGCTCTGAAGAGTTCGAT-3′	5′-TGTGAGCCTTCTCCGTCTTT-3′
BIP	NM_213058	5′-AAGAGGCCGAAGAGAAGGAC -3′	5′-AGCAGCAGAGCCTCGAAATA -3′
CHOP	XM_005166171	5′-AAGGAAAGTGCAGGAGCTGA -3′	5′-TCACGCTCTCCACAAGAAGA- 3′
Beclin-1	NM_200872	5′-AGAGCATTGAGACAAAGCGTGAA -3′	5′-TCTGCCAAGGCGGAAGTTATT-3′
ATG12	BC154046	5′-TTCATCTCACGCTTCCTCAA-3′	5′-CGTCACTTCCGAAACACTCA-3′
ATG3	NM_200022	5-′GGCTGTTTGGATATGATGAG-3′	5′-AGCAGGTGGAGGGAGATTAG- 3′
IL-1β	AY340959	5′-TGGCGAACGTCATCCAAG-3′	5′-GGAGCACTGGGCGACGCATA-3′
TNF-α	AY427649	5′-GCTTATGAGCCATGCAGTGA-3′	5′-TGCCCAGTCTGTCTCCTTCT-3′
NF-κB	BC162402	5′-GAAGCATTCAGGCTCGGTGA-3′	5′-CAGGTCTGTCGGTCCCTTTC-3′
EFL1-alpha	AY422992	5′-TACTTCTCAGGCTGACTGTG-3	5′-ATCTTCTTGATGTATGCGCT-3′

### Western bloting

10–15 zebrafish larvae split from head to tail were lysed on ice in RIPA buffer (Cell Signaling Technology) supplemented with protease inhibitors (Roche). Quantified protein lysates were separated on a 10 % SDS-PAGE gels, electrotransferred onto polyvinylidene fluoride (Millipore) membranes, blocked with 5 % bovine serum albumin for 1 h at room temperature, and immunoblotted with primary antibodies CHOP (G6916, Sigma) (1:400), BIP (MB0050,Bioworld) (1:1000), IL-1β (16806-1-AP, Prointech) (1:1000),β-actin (sc-8432, Santa Cruz Biotechnology) (1:1000) overnight at 4 C. After the incubation with the corresponding secondary antibodies conjugated to horseradish peroxidase, the signals of the membranes were detected by enhanced chemiluminescence Western Blotting Substrate (Pierce, Rockford). The band intensity of Western blotting and the normalization were analyzed using the ImageJ program (NIH, Bethesda,MD).

### Statistics

All data are presented as means ± standard error of the mean. These groups were tested for the effects of feeding quantity, duration of feeding, and/or their interactions by two-way analysis of variance. When the interaction and/or the main effects were significant, the group means were compared further using the Bonferroni’s multiple comparison tests. Differences between 2 groups were examined for statistical significance by Student’s *t* –test. For multiple comparisons, one-way ANOVA followed by Bonferroni-Dunn multiple-comparison procedure was used. *P* < 0.05 was considered statistically significant. All statistical analyses were performed using GraphPad Prism 5.00 for Windows (GraphPad, San Diego, CA, USA).

## Results

### Zebrafish larvae overfed diets remarkably developed hepatic steatosis

**Fig. 1:**
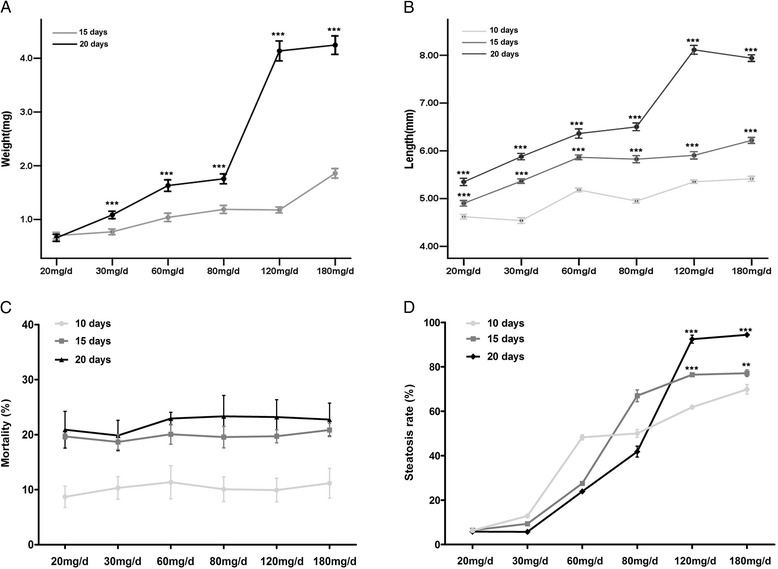
Effects of quantity and duration of feeding on growth and incidence of hepatic steatosis and mortality of zebrafish larvae. Body weight (**a**) and length (**b**) of zebrafish larvae were measured in different feeding groups for different feeding time (*n* = 80–91). **c** and **d** represent the effect of the quantity of feeding on mortality and hepatic steatosis rate in zebrafish larvae fed for different feeding time, respectively (*n* = 3). Data are expressed as mean ± SEM, **P < 0.01, ***P < 0.001

**Fig. 2:**
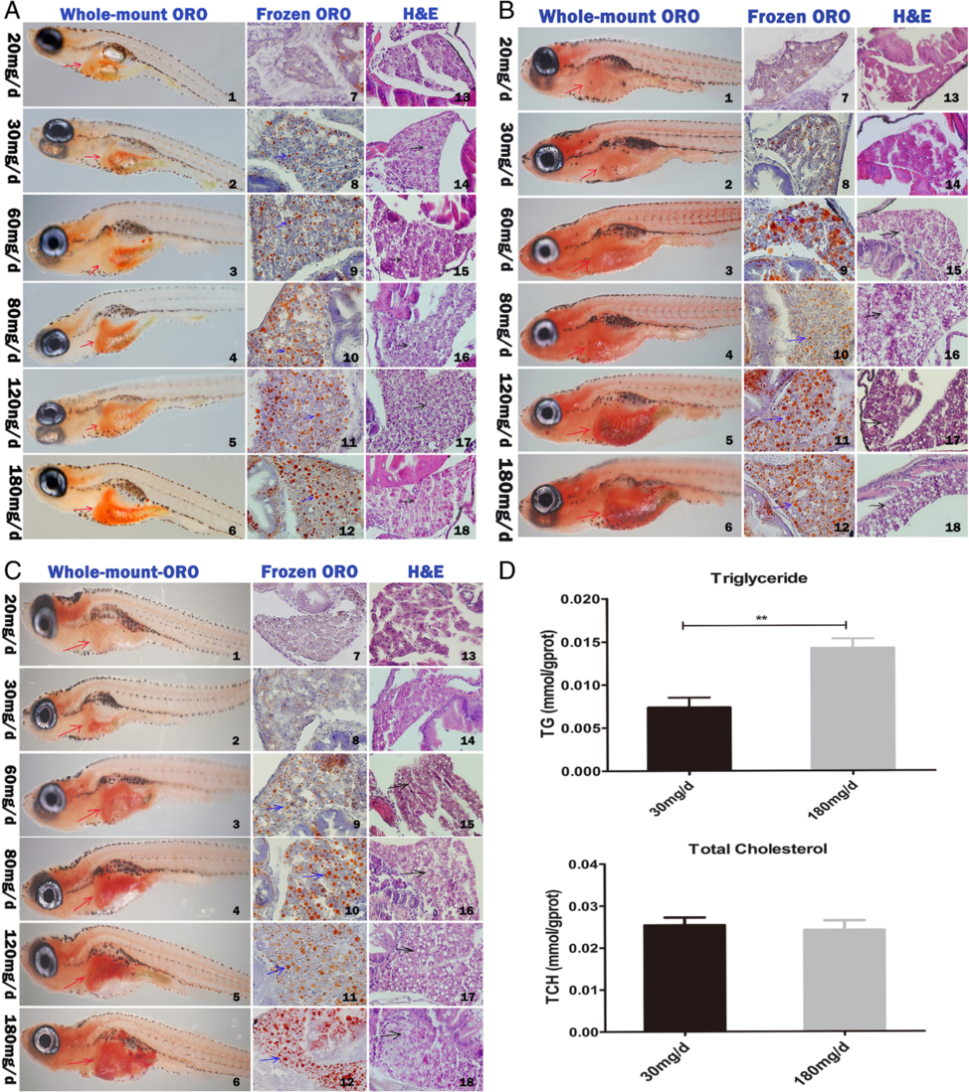
The liver histological changes of zebrafish larvae fed in different quantity of feeding and lipid contents in zebrafish. The histological change of liver in zebrafish larvae fed for 10 (**a**), 15 (**b**), and 20 (**c**) days was analyzed using Whole-mount oil red O staining (whole-mount ORO) (panels 1–6) (magnification × 32), frozen section oil red O staining (frozen ORO) (panels 7–12) (magnification × 400) and H&E staining (panels 13–18) (magnification × 400). Red arrows indicate zebrafish liver, blue arrows indicate lipid droplets, black arrows indicate vacuole lipid droplets. **d** Changes of triglyceride (TG) and total cholesterol (TCH) of zebrafish larvae fed for 20 days in 30 mg/d and 180 mg/d feeding group, Data are represented as mean ± SEM, *n* = 3. **P < 0.01 by Student’s *t* test

Zebrafish have the feature of food preference. To understand the association between the quantity and duration of feeding and hepatic steatosis of zebrafish larvae, we observed the changes of larval growth and hepatic steatosis under different quantity and duration of feeding conditions. Figure 1 shows that the body weight (Fig. 1a) and length (Fig. 1b) of larvae does-dependently increased with the increased amount of feeding, and the body weight of larvae fed for 20 days was significantly increased than that fed for 15 days (Fig. 1a). Similarly, the body length of larvae fed for 20 days was markedly increased than that fed for 10 and 15 days (Fig. 1b). However, the mortality of larvae between the different feeding groups had no significant difference (Fig. 1c); The death rate of larvae fed for 15 and 20 days was significantly higher than that fed for 10 days, but it was not a significant change between larvae fed for 15 and 20 days (Fig. 1c). The data suggested that the mortality of larvae could be disassociated with the amount of feeding and depend on inherent quality of zebrafish larvae per se. The incidence of hepatic steatosis of larvae was gradually increased with the increased amount of feeding when estimated by whole-mount oil red O (whole-mount ORO) staining, and it was 92.4 and 94.4 % in larva fed on 120 mg and 180 mg per day for 20 days, respectively (Fig. 1d). Then we evaluated the degree of hepatic steatosis of larvae in different quantity and duration of feeding by using histological methods. The results of whole-mount ORO and Frozen ORO and HE staining showed that the liver in zebrafish larvae fed for 10 days had lipid droplets accumulation (Fig. 2a) and the droplets became more intensive and bigger when larva fed for 15 and 20 days (Fig. 2b,c). However, the liver in zebrafish larvae fed 20 or 30 mg/d basically haven’t lipid accumulation, indicating this amount of feeding can maintain the normal need of energy for zebrafish larva. And the larvae fed 120 or 180 mg/d shown considerable lipid accumulation in the liver, which suggested that in this condition zebrafish larvae have surpassed the need of energy. Further, we examined the level of triglyceride (TG) and total cholesterol (TCH) in zebrafish larvae. The results revealed that the content of TG was significantly higher in larvae fed 180 mg/d (0.0142 ± 0.0011 mmol/gprotein) compared to larvae fed 30 mg/d (0.0073 ± 0.0012 mmol/gprotein) for 20 days (Fig. 2d), and there was no difference in the level of TCH between the two groups (Fig. 2d). Thus, these data demonstrated that zebrafish larvae were quite readily to have hepatosteatosis under the overfeed dietary conditions and we successfully established an diet-induced model of fatty liver in zebrafish larvae and provided a platform for screening therapies drugs and studying mechanism for NALFD.

### Caffeine attenuated hepatic lipid accumulation in overfed zebrafish larvae

**Fig. 3:**
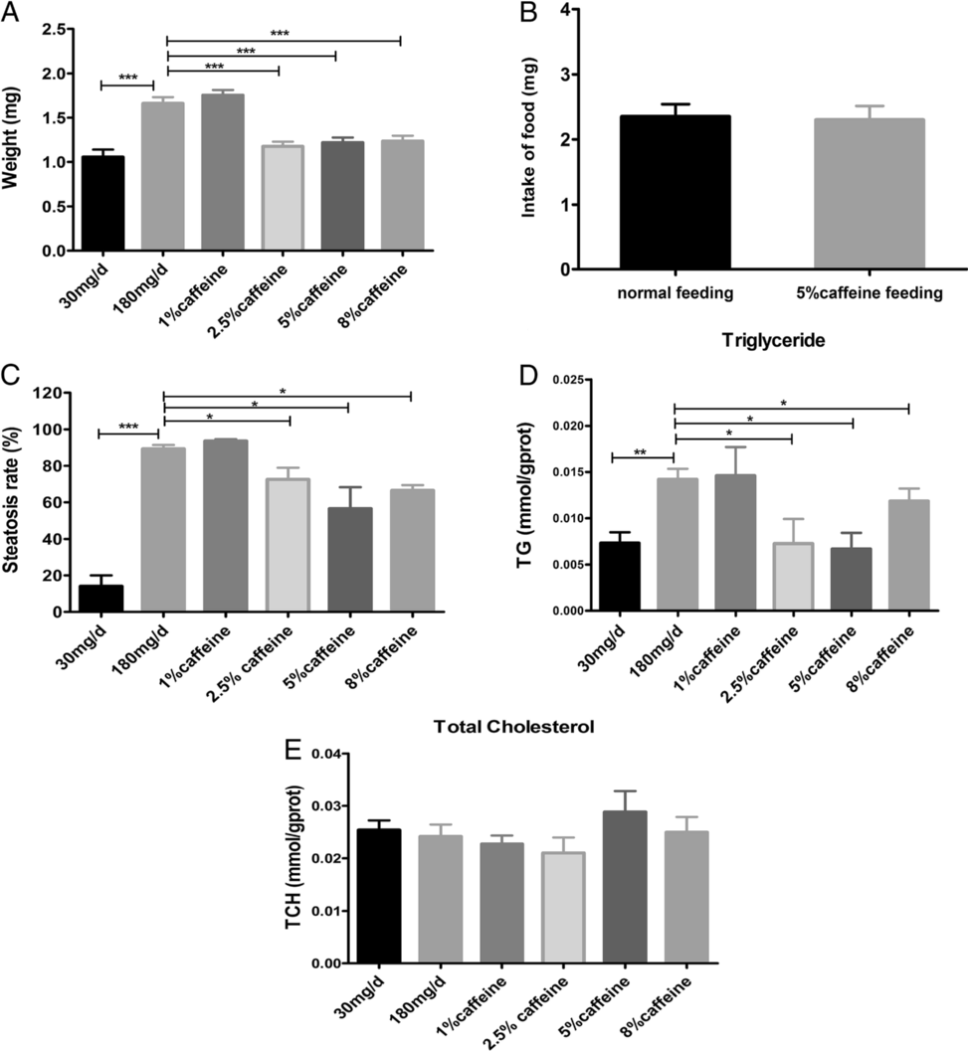
Effects of Caffeine on hepatic steatosis and lipid contents in overfed zebrafish larvae for 20 days. **a** The effects of caffeine on weight in zebrafish larvae overfed for 20 days. *n* = 2 clutches per group, *n* = 79–86 per clutch. **b** The food intake of 15 larvae in normal feeding and 5 % caffeine feeding group (*n* = 4). **c** Hepatic steatosis rate in zebrafish larvae overfed for 20 days (*n* = 3). The contents of triglyceride (**d**) and total cholesterol (**e**) were measured in zebrafish larvae overfed with different concentration of caffeine for 20 days (*n* = 3). Data is expressed as mean ± SEM. *P < 0.05, **P < 0.001, ***P < 0.001 by one-way ANOVA

**Fig. 4:**
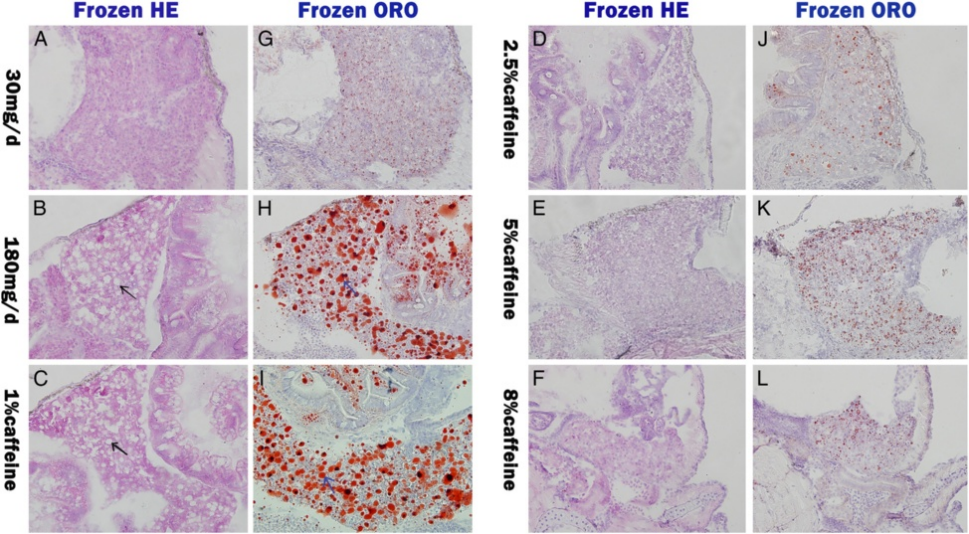
Effects of caffeine on hepatic lipid accumulation in zebrafish larvae overfed for 20 days. Frozen section hematoxylin and eosin staining (Frozen H&E) of liver sections in 30 mg/d (**a**), 180 mg/d (**b**), 1 % caffeine (**c**), 2.5 % caffeine (**d**), 5 % caffeine (**e**), and 8 % caffeine (**f**) feeding zebrafish larvae. Black arrow indicate lipid droplet. Frozen Oil red O staining (Frozen ORO) of liver sections in 30 mg/d (**g**), 180 mg/d (**h**), 1 % caffeine (**i**), 2.5 % caffeine (**j**), 5 % caffeine (**k**), and 8 % caffeine (**l**) feeding zebrafish larvae. Blue arrow indicate lipid droplet

Many epidemiological and clinical evidences have demonstrated that coffee and caffeine have protective effects on the chronic liver diseases. So we investigated whether caffeine can reduce hepatic steatosis in overfeeding zebrafish larvae or not. First, we evaluated the effect of caffeine on the body weight of zebrafish. We found that body weight of zebrafish was remarkably reduced in 2.5, 5 and 8 % caffeine groups compared with the model group (180 mg/d), but there was no significant difference in 1 % caffeine group compared to the model group (Fig. 3a). Meanwhile, the food intake of larvae have no significant difference between normal feeding and 5 % caffeine feeding group (Fig. 3b), suggesting that caffeine have no influence on the appetite of zebrafish. As like the body weight, the hepatic steatosis rate (Fig. 3c) and the TG content (Fig. 3d) of zebrafish significantly decreased in 2.5, 5 and 8 % caffeine group and there was no difference between 1 % caffeine and model group, and the content of TCH have no significance between all groups (Fig. 3e). Then frozen consecutive serial 8 μm sections of a zebrafish larva in different groups were collected on different slides, and one slide was stained with hematoxylin and eosin (HE), while the other was stained with oil red O (ORO) staining to observe lipid droplets. The amount and size of lipid droplets in the liver of zebrafish was markedly reduced in 2.5, 5 and 8 % caffeine group compared with 1 % caffeine and model group (Fig. 4). Overall, our data showed that caffeine can alleviate hepatic steatosis in overfed zebrafish larvae.

### Caffeine got involved in modulating the expression of genes associated with lipid metabolism pathways,endoplasmic reticulum (ER) stress and hepatic inflammatory cytokine

**Fig. 5:**
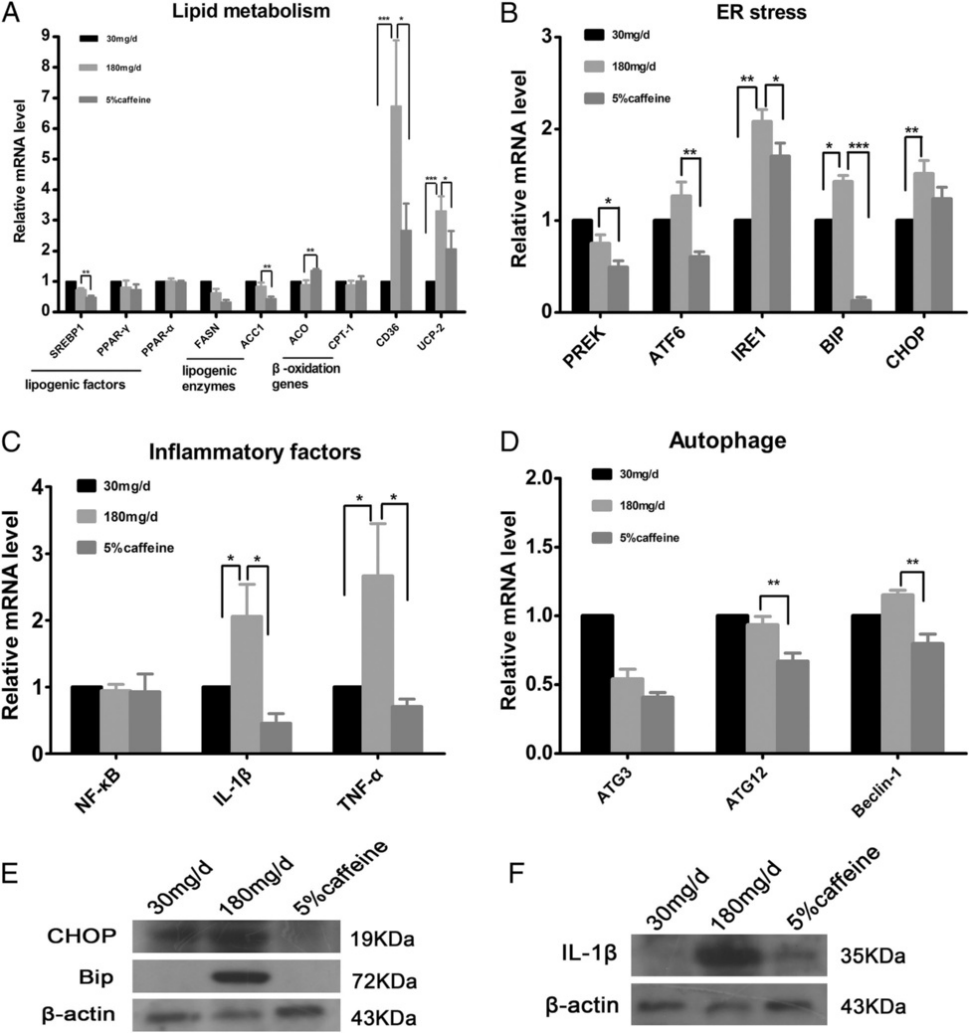
Effects of caffeine on the expression of selected genes in livers of zebrafish larvae overfed for 20 days. The relative mRNA expression of genes involved in lipid metabolism (**a**), ER stress (**b**), inflammatory factors (**c**), and autophage (**d**) in control (30 mg/d) and 5 % caffeine group compared with gene expression in model group (180 mg/d) by qRT-PCR. Expression analysis of the selected genes using cDNA prepared from liver of zebrafish larvae (*n* = 20) in each group. Data is expressed in mean ± SEM, *n* = 5. Protein expression of ER stress (**e**) and inflammatory factor (**f**) was examined by western blot in caffeine treatment and model group zebrafish larvae. * *P* < 0.05, * *P < 0.01, * * *P < 0.001 by one-way ANOVA

To investigate the mechanism by which caffeine reduced hepatic lipid accumulation, we examined the expression levels of genes involved in lipid metabolism (Fig. 5a). We first examined whether or not decreased lipid accumulation in the liver of caffeine treatment group occurred preferentially by dietary fat and uptake through receptor-mediated endocytosis. The level of gene expression of fatty acid transport protein exhibited notably downregulation in 5 % caffeine group compared with model group, including fatty acid translocase (FAT)/CD36 (Fig. 5a). The mRNA levels of uncoupling protein −2(UCP-2) and gene involved in hepatic lipogenesis transcription factor as sterol regulatory element binding transcription factor 1(SREBP1) was decreased. The level of gene expression of key lipogenic enzymes involved in fatty acid synthesis was significantly downregulated, including acetyl-CoA carboxylase 1 (ACC1). Although the mRNA level of fatty acid synthase (FASN) have no statistical difference between model group and caffeine treatment group, its level also reduced in zebrafish larvae fed on 5 % caffeine. But the mRNA level of gene acyl-CoA oxidase (ACO) involved in fatty acid β-oxidation was significantly increased in 5 % caffeine group (Fig. 5a). Many evidences have exhibited that ER stress is involved in the development of fatty liver and can promote the progression of NALFD. We explored whether caffeine can impact on the function of ER to perform protective effects on hepatocytes in overfed zebrafish larvae (Fig. 5b). We found that the mRNA level of genes involved in ER stress were significantly upregulated in the liver of model group compared to control group (30 mg/d), including IRE1, BIP, CHOP. After caffeine treatment, the gene expression level of IRE1 and BIP were decreased, and the mRNA level of genes ATF6 and PERK were also remarkably reduced in the liver of caffeine treatment larvae (Fig. 5b). Further, western blot analysis showed that the level of Bip and CHOP increased in model group compared to control group and caffeine treatment resulted in a marked reduction of the expression of them (Fig. 5e). These results indicate that caffeine could improve ER stress induced by overfeeding in zebrafish larvae. Next, we examined the expression level of inflammatory cytokine genes to confirm whether caffeine can regulate inflammatory response. As Fig. 5c showed that the inflammatory cytokines, including IL-1beta and TNF-alpha, were markedly increased in the liver of model group compared with control group. The mRNA level of IL-1beta and TNF-alpha were significantly decreased in 5 % caffeine group compared to model group. The protein level of IL-1beta also decreased in caffeine treatment group as evidenced by western blot (Fig. 5f). No significant difference was observed in the expression of NF-κB. In addition, the mRNA level of ATG12 and Beclin-1, which is implicated in autophagy, were significantly decreased in the liver of 5 % caffeine treatment group (Fig. 5d). Taken together, these results indicated that caffeine may inhibit lipogenesis and lipid transport, and increase lipid oxidation, and probably improve ER stress and reduce hepatic inflammatory response to ameliorate hepatic lipid accumulation in zebrafish larvae.

## Discussion

In present study, we examined the efficacy of caffeine in fatty liver zebrafish larvae via successful establishment of a diet-induced zebrafish larva model for NAFLD. Our results demonstrated that juvenile zebrafish is readily to be induced to pronounced hepatic lipid accumulation undergoing an overfeeding regimen for 20 days. The rate of hepatic steatosis could reach up to 90 % in overfed zebrafish larvae. In addition, we demonstrated that caffeine can attenuate hepatic lipid accumulation in diet-induced fatty liver zebrafish through inhibition of fatty acid uptake and lipogenesis and improvement of ER function, and attenuation of inflammation response.

As a new model of studying human diseases, zebrafish have got more and more attentions for researchers in the pathogenesis of investigating human conditions and drugs screening, the main causes of which is that zebrafish possess plenty of merits such as the similarity of its genome with humans, the tractability of its genetics, short breeding period, and low cost and convenience of feeding. Many studies have reported that zebrafishes were exploited to identify the molecular mechanisms of nonalcoholic fatty liver diseases [11, 13, 14, 40, 41] and performed associated drugs screening [42–44], which shows the great value of its application in human diseases. This study shown that zebrafish larvae in juvenile have significant lipid accumulation in the liver over the increasing quantity and duration of feeding via the observation of morphological and histological methods in zebrafish. The percentage of hepatic steatosis of zebrafish larvae reached up to 90 % in the groups fed on 120 mg/d or 180 mg/d for 20 days, and Whole-mount oil red O staining and HE staining showed that macrovesicular lipid droplets presented in liver after fish fed for 20 days. However, zebrafish larvae fed with 20 mg/d or 30 mg/d hardly have lipid droplets in liver and there is no significant difference in the mortality of larvae between all groups. The level of triglyceride of larvae in the model group is significantly higher than that in the controlg group, and the mRNA level of inflammatory cytokines IL-1beta and TNF-alpha are upregulated in the model group. These data suggested that zebrafish larvae are readily to develop fatty liver and have a trend toward the progression of NASH under the overfeeding conditions (180 mg/d). Considering that zebrafish are in the period of growth and development and the prevalence of NAFLD in children and adolescent is increasing at present, this model provides an opportunity and reference for studying NAFLD in children and adolescent.

We demonstrated that caffeine can reduce hepatic lipid accumulation in zebrafish, which was coordinated with the results of previous researchers studying the effects of caffeine in human [27, 29], rats [38] and hepatocytes [39]. Although caffeine displayed the does-dependence association with reduced lipid level of HepG2 cell in vitro [39], this relationship was not observed in our study in zebrafish. Many studies have demonstrated that caffeine exist the effect of anti-obesity through reduction of the size of adipose tissue and amount of adipocytes [45, 46] and increase of heat production of adipose tissue and the basal metabolic rate of body [47] in mice. Moreover, in vitro study suggested that caffeine can inhibit the development and differentiation of adipocyte through inhibition of adipogenic related factors [48]. These roles of caffeine maybe explain the reduction of body weight in caffeine treatment zebrafish in our research. However, further researches are required to investigate whether caffeine have the role of inhibition of development and differentiation of adipocyte in vivo. Our data shown that low concentration of caffeine (<1 %) didn’t change lipid accumulation in liver of zebrafish and moderate concentration of caffeine (2.5–8 %) exhibited the antisteatotic effect, which was consistent with epidemiological and clinical evidences that moderate caffeine intake have a hepatoprotective effect on NAFLD and fibrosis/cirrhosis [34, 49] with consumption of more than three cups of coffee per day. The mortality of larvae markedly increased when caffeine concentration reached up to 10 % (data not shown), indicating that high concentration caffeine may present toxicity in zebrafish larvae probably because of the induction of cell apoptosis in high caffeine concentration [50–52]. So maybe it is a good advice to drink a moderate coffee in daily life.

Regarding to the molecular mechanism of caffeine alleviating hepatic lipid accumulation, we determined the expression of genes associated with lipid metabolism. Our results shown downregulation of transcription factor SREBP1, which control the lipogenesis transcriptionally, and its downstream genes ACC1 and FASN that are key enzymes in the lipid synthesis, and upregulation of ACO gene which involve in lipid beta-oxidation. This result is consistent with previous findings of Hai Yan Quan [39], suggesting that caffeine can inhibit de novo lipogenesis and enhance lipid oxidation in liver. Fatty acid translocase (FAT/CD36) is a membrane protein participated in fatty acid intake of hepatocytes. Clinical evidence have demonstrated that CD36 is significantly increased in fatty liver and NASH patients [53], indicating of the association of the development of NAFLD with increased input of fatty acid in hepatocytes. Our data exhibited the reduced mRNA level of CD36 in caffeine treatment larvae, demonstrating that caffeine may be exert antisteatotic effect through reduction of fatty acid uptake. Endoplasmic reticulum (ER) stress is an adaptive response due to excessive accumulation of unfolded responsive protein in cells. There have numerous evidences that ER stress can contribute to fatty liver, promote the progression of NASH [54] and the development of obesity,and activate the inflammatory signalling pathway mediated by NF-κB-IκB kinase (IKK) and JNK signalling [55, 56]. Recent reports have shown that caffeine can improve leptin-resistance of neuron by reduced ER stress via attenuation of activation of IRE-1 and PERK [57] and that coffee can show anti-injury role by reducing the level of IL-6 and TNF-alpha in the Long-Evans Cinnamon rat [58]. In present study, we found that caffeine markedly reduced expression of genes associated with ER stress including IRE-1, PERK, ATF6, CHOP and BIP, indicating that caffeine may improve ER stress to protect liver cell. Simultaneously, caffeine also decreased the expression level of inflammatory cytokines such as IL-1beta and TNF-alpha. This is likely to be caused by improvement of ER stress, because ER stress can activate the signalling pathway of inflammation response. Although our data shown that caffeine may have an effect on enhancement of the adaptive ability of zebrafish in response to stress at the level of transcription, there need more studies to research the precise molecular mechanism by which caffeine exert hepatoprotective effect in NAFLD.

## Conclusions

The present experiment shows that zebrafish larvae are quite readily to develop hepatic steatosis by the overfeeding regimen. We demonstrated that caffeine have an antisteatotic and hepatoprotective effect on fatty liver in zebrafish, maybe through reduction of fatty acid uptake and lipogenesis, enhancement of lipid beta-oxidation, improvement of ER stress and attenuation of inflammation response. Thus caffeine can become a potential drug for NAFLD treatment and zebrafish model of NAFLD provide a cost and convenient platform for drug screening.

## Abbreviations

Dpf: Days post-fertilization
PPAR-γ: Peroxisome proliferator-activated receptor gamma
SREBP1: Sterol regulatory element binding protein1
PPAR-α: Peroxisome proliferator activated receptor alpha
ACC1: Acetyl-CoA carboxylase 1
FASN: Fatty acid synthase
CD36: Fatty acid translocase
UCP-2: Uncoupling protein −2
ACO: Acyl-CoA oxidase
CPT-1: Carnitine palmitoyltransferase1
PERK: PRKR-like endoplasmic reticulum kinase
IRE-1: Inositol-requiring enzyme 1
ATF-6: Activating transcription factor-6
CHOP: DNA damage-inducible transcript 3
BIP: Binding immunoglobulin protein
IL-1β: Interleukin-1 beta
TNF-α: Tumor necrosis factor alpha
ATG-3: Autophagy gene 3
ATG-12: Autophagy related 12 homolog
NAFLD: Non-alcoholic fatty liver disease
NASH: Non-alcoholic steatohepatitis
ER: Endoplasmic reticulum
